# Supplementary material for: Decadal changes and delayed avian species losses due to deforestation in the northern Neotropics
Source: PeerJ. 2013 Oct 8;1:e179. doi: 10.7717/peerj.179 (PMC3796372; doi:10.7717/peerj.179)
Supplement: Appendix S1 [file peerj-01-179-s001.pdf]

Appendix. Species, number of captures (captures per 1000 net hours), and total captures by sampling period. Excludes obligate seasonal migrants.

Species	(1)	(2)	(3)	(4)	(5)	(6)	(7)	(8)	Total
Micrastur ruficollis	3 (0.088)	0	0	0	0	0	0	0	3
Micrastur semitorquatus	0	0	0	0	0	0	1 (0.119)	0	1
Tinamus major	0	0	0	1 (0.079)	0	0	0	0	1
Crypturellus boucardi	0	0	1 (0.232)	0	0	0	0	0	1
Columbina passerina	0	0	0	1 (0.079)	0	0	0	0	1
Columbina talpacoti	0	16 (0.438)	0	3 (0.238)	3 (0.073)	0	0	0	22
Claravis pretiosa	0	3 (0.082)	0	0	0	1 (0.044)	0	0	4
Leptotila verreauxi	0	1 (0.027)	0	12 (0.952)	6 (0.146)	1 (0.044)	0	0	20
Leptotila plumbeiceps	12 (0.353)	5 (0.137)	2 (0.464)	7 (0.555)	5 (0.122)	12 (0.533)	4 (0.476)	1 (0.433)	36
Geotrygon montana	30 (0.883)	6 (0.164)	1 (0.232)	1 (0.079)	3 (0.073)	15 (0.666)	9 (1.072)	0	65
Crotophaga sulicirostris	1 (0.029)	6 (0.164)	0	5 (0.397)	1 (0.024)	1 (0.044)	0	0	14
Piaya cayana	0	2 (0.055)	0	2 (0.159)	1 (0.024)	0	2 (0.238)	0	7
Glaucidium brasilianum	5 (0.147)	7 (0.192)	2 (0.464)	3 (0.238)	1 (0.024)	1 (0.044)	0	0	19
Ciccaba virgata	1 (0.029)	3 (0.082)	1 (0.232)	0	1 (0.024)	0	0	0	6
Nyctidromus albicollis	1 (0.029)	0	0	0	1 (0.024)	2 (0.089)	0	0	4

Species	(1)	(2)	(3)	(4)	(5)	(6)	(7)	(8)	Total
Phaethornis longirostris	186 (5.474)	161 (4.410)	18 (4.176)	68 (5.395)	64 (1.556)	119 (5.287)	22 (2.621)	3 (1.298)	641
Phaethornis striigularis	22 (0.648)	21 (0.575)	1 (0.232)	6 (0.476)	2 (0.049)	1 (0.044)	3 (0.357)	0	56
Heliomaster longirostris	1 (0.029)	0	0	0	0	0	0	0	1
Campylopterus exellens	44 (1.295)	29 (0.794)	7 (1.624)	31 (2.459)	6 (0.146)	29 (1.288)	9 (1.072)	0	155
Campylopterus hemileucurus	83 (2.443)	127 (3.478)	0	48 (3.808)	55 (1.337)	95 (4.221)	15 (1.787)	0	423
Florisuga mellivora	4 (0.118)	0	0	0	0	0	0	0	4
Anthracothorax prevostii	0	9 (0.246)	0	0	0	0	2 (0.238)	0	11
Hylocharis eliciae	1 (0.029)	1 (0.027)	0	0	0	0	0	0	2
Amazilia candida	115 (3.385)	142 (3.889)	2 (0.464)	25 (1.983)	12 (0.292)	29 (1.288)	23 (2.740)	2 (0.865)	350
Amazilia tzacatl	7 (0.206)	31 (0.849)	0	1 (0.079)	1 (0.024)	2 (0.089)	1 (0.119)	0	43
Amazilia yucatanensis	0	0	0	2 (0.159)	0	0	0	0	2
Chlorostilbon canivetii	0	1 (0.027)	0	0	0	0	0	0	1
Colibri thalassinus	0	1 (0.027)	0	0	0	0	2 (0.238)	0	3
Trogon collaris	1 (0.029)	0	1 (0.232)	5 (0.397)	2 (0.049)	7 (0.311)	2 (0.238)	1 (0.433)	19
Trogon massena	0	1 (0.027)	0	1 (0.079)	0	1 (0.044)	0	0	3

Species	(1)	(2)	(3)	(4)	(5)	(6)	(7)	(8)	Total
Trogon violaceus	0	0	0	0	0	0	1 (0.119)	0	1
Trogon melanocephalus	0	0	0	0	0	0	1 (0.119)	0	1
Pteroglossus torquatus	10 (0.294)	6 (0.164)	0	2 (0.159)	2 (0.049)	3 (0.133)	4 (0.476)	0	27
Hylomanes momotula	0	0	2 (0.464)	8 (0.635)	7 (0.170)	6 (0.267)	0	0	23
Momotus momota	4 (0.118)	3 (0.082)	3 (0.696)	9 (0.714)	2 (0.049)	19 (0.844)	10 (1.191)	1 (0.433)	44
Chloroceryle aenea	2 (0.059)	9 (0.246)	0	0	0	0	0	0	11
Chloroceryle americana	1 (0.029)	0	0	0	0	1 (0.044)	0	0	2
Centurus aurifrons	2 (0.059)	8 (0.219)	0	10 (0.793)	2 (0.049)	4 (0.178)	0	1 (0.433)	11
Centurus pucherani	3 (0.088)	4 (0.110)	0	1 (0.079)	0	0	0	0	7
Piculus rubiginosus	2 (0.059)	4 (0.110)	0	3 (0.238)	0	1 (0.044)	3 (0.357)	0	7
Venilornis fumigatus	15 (0.441)	15 (0.411)	1 (0.232)	1 (0.079)	1 (0.024)	1 (0.044)	2 (0.238)	1 (0.433)	37
Celeus castaneus	7 (0.206)	8 (0.219)	0	0	0	1 (0.044)	0	0	16
Dryocopus lineatus	0	1 (0.027)	0	0	0	0	0	0	1
Lepidocolaptes souleyetii	4 (0.118)	3 (0.082)	1 (0.232)	1 (0.079)	0	0	0	0	2
Xiphorhynchus flavigaster	1 (0.029)	1 (0.027)	7 (1.624)	12 (0.952)	9 (0.219)	9 (0.400)	15 (1.787)	6 (2.595)	60

Species	(1)	(2)	(3)	(4)	(5)	(6)	(7)	(8)	Total
Sittasomus griseicapillus	39 (1.148)	14 (0.383)	2 (0.464)	5 (0.397)	4 (0.097)	6 (0.267)	4 (0.476)	0	74
Glyphorynchus spirurus	50 (1.472)	16 (0.438)	0	0	0	0	0	0	66
Dendrocolaptes certhia	14 (0.412)	4 (0.110)	2 (0.464)	5 (0.397)	3 (0.073)	2 (0.089)	1 (0.119)	1 (0.433)	14
Dendrocincla anabatina	51 (1.501)	22 (0.603)	6 (1.392)	6 (0.476)	10 (0.243)	4 (0.178)	4 (0.476)	3 (1.298)	106
Xenops minutus	36 (1.060)	27 (0.739)	1 (0.232)	7 (0.555)	5 (0.122)	6 (0.267)	1 (0.119)	1 (0.433)	21
Synallaxis erythrothorax	0	6 (0.164)	0	0	0	0	0	0	6
Ramphocaenus melanurus	3 (0.088)	12 (0.329)	2 (0.464)	5 (0.397)	2 (0.049)	0	4 (0.476)	0	28
Anabacerthia variegaticeps	0	0	0	5 (0.397)	5 (0.122)	8 (0.355)	0	0	18
Automolus ochrolaemus	10 (0.294)	8 (0.219)	2 (0.464)	4 (0.317)	3 (0.073)	7 (0.311)	1 (0.119)	0	35
Taraba major	0	1 (0.027)	0	0	0	0	0	0	1
Thamnophilus doliatus	0	2 (0.055)	0	5 (0.397)	5 (0.122)	4 (0.178)	0	0	16
Formicarius analis	4 (0.118)	0	0	0	0	0	0	0	4
Grallaria guatimalensis	1 (0.029)	0	1 (0.232)	0	0	0	0	0	2
Ornithion semiflavum	0	7 (0.192)	1 (0.232)	3 (0.238)	0	6 (0.267)	0	0	10
Mionectes oleagineus	149 (4.385)	92 (2.520)	36 (8.353)	156 (#####)	97 (2.358)	140 (6.220)	27 (3.216)	4 (1.730)	701

Species	(1)	(2)	(3)	(4)	(5)	(6)	(7)	(8)	Total
Platyrinchus cancrominus	144 (4.238)	76 (2.082)	14 (3.248)	18 (1.428)	23 (0.559)	18 (0.800)	21 (2.501)	11 (4.758)	325
Elaenia flavogaster	4 (0.118)	0	0	0	0	0	0	0	4
Onychorhynchus cornonatus	10 (0.294)	3 (0.082)	1 (0.232)	0	0	0	0	0	14
Leptopogon amaurocephalus	20 (0.589)	6 (0.164)	3 (0.696)	7 (0.555)	3 (0.073)	3 (0.133)	0	0	45
Tolmomyias sulphurescens	18 (0.530)	12 (0.329)	1 (0.232)	11 (0.873)	9 (0.219)	6 (0.267)	3 (0.357)	1 (0.433)	61
Myiobius sulphureipygius	99 (2.914)	39 (1.068)	1 (0.232)	3 (0.238)	6 (0.146)	2 (0.089)	0	0	17
Rhynchocyclus brevirostris	63 (1.854)	44 (1.205)	2 (0.464)	24 (1.904)	18 (0.438)	36 (1.599)	1 (0.119)	0	188
Contopus cinereus	0	1 (0.027)	0	0	0	0	0	0	1
Myiarchus tuberculifer	1 (0.029)	0	0	8 (0.635)	4 (0.097)	5 (0.222)	0	1 (0.433)	19
Myiarchus tyrannulus	0	11 (0.301)	0	0	0	0	0	0	11
Attila spadiceus	51 (1.501)	28 (0.767)	5 (1.160)	12 (0.952)	8 (0.194)	7 (0.311)	5 (0.596)	0	116
Pipra mentalis	50 (1.472)	11 (0.301)	18 (4.176)	34 (2.697)	21 (0.510)	22 (0.977)	8 (0.953)	1 (0.433)	165
Megarhynchus pitangua	2 (0.059)	3 (0.082)	0	3 (0.238)	0	1 (0.044)	1 (0.119)	1 (0.433)	6
Pitangus sulphuratus	1 (0.029)	0	0	2 (0.159)	0	0	0	0	3
Myiozetetes similis	2 (0.059)	0	0	1 (0.079)	0	0	0	0	3

Species	(1)	(2)	(3)	(4)	(5)	(6)	(7)	(8)	Total
Pachyramphus aglaiae	6 (0.177)	4 (0.110)	0	7 (0.555)	1 (0.024)	1 (0.044)	4 (0.476)	0	13
Tityra inquisitor	1 (0.029)	0	0	0	0	0	0	0	1
Tityra semifasciata	1 (0.029)	0	1 (0.232)	2 (0.159)	2 (0.049)	0	4 (0.476)	0	10
Cotinga amabilis	1 (0.029)	0	0	0	0	0	0	0	1
Schiffornis turdina	0	1 (0.027)	0	0	0	0	0	0	1
Polioptila plumbea	1 (0.029)	0	0	0	0	0	0	0	1
Campylorhynchus zonatus	0	0	1 (0.232)	0	1 (0.024)	0	0	0	2
Troglodytes aedon	8 (0.235)	7 (0.192)	1 (0.232)	0	1 (0.024)	2 (0.089)	1 (0.119)	0	20
Henicorhina leucosticta	100 (2.943)	59 (1.616)	10 (2.320)	19 (1.507)	110 (2.674)	19 (0.844)	8 (0.953)	1 (0.433)	167
Thryothorus maculipectus	59 (1.737)	70 (1.917)	6 (1.392)	12 (0.952)	11 (0.267)	12 (0.533)	19 (2.263)	3 (1.298)	192
Myadestes unicolor	3 (0.088)	0	0	0	0	0	0	0	3
Catharus mexicanus	0	0	0	0	1 (0.024)	0	0	0	1
Turdus assimilis	7 (0.206)	20 (0.548)	4 (0.928)	8 (0.635)	4 (0.097)	8 (0.355)	6 (0.715)	0	26
Turdus grayi	26 (0.765)	21 (0.575)	7 (1.624)	32 (2.539)	5 (0.122)	22 (0.977)	14 (1.668)	1 (0.433)	81
Vireolanius pulchellus	0	0	0	1 (0.079)	0	0	0	1 (0.433)	2

Species	(1)	(2)	(3)	(4)	(5)	(6)	(7)	(8)	Total
Hylophilus decurtatus	34 (1.001)	40 (1.096)	0	9 (0.714)	4 (0.097)	2 (0.089)	3 (0.357)	1 (0.433)	93
Hylophilus ochraceiceps	129 (3.797)	54 (1.479)	9 (2.088)	3 (0.238)	4 (0.097)	3 (0.133)	9 (1.072)	2 (0.865)	213
Myioborus miniatus	0	0	0	2 (0.159)	0	0	0	0	2
Geothlypis poliocephala	0	10 (0.274)	0	4 (0.317)	0	2 (0.089)	3 (0.357)	0	19
Basileuterus culicivorus	0	0	12 (2.784)	43 (3.411)	25 (0.608)	54 (2.399)	8 (0.953)	3 (1.298)	145
Basileuterus rufifrons	4 (0.118)	17 (0.466)	1 (0.232)	3 (0.238)	2 (0.049)	11 (0.489)	2 (0.238)	2 (0.865)	42
Coereba flaveola	2 (0.059)	11 (0.301)	3 (0.696)	2 (0.159)	2 (0.049)	4 (0.178)	0	0	11
Euphonia affinis	0	1 (0.027)	0	0	0	0	0	0	1
Euphonia gouldi	29 (0.854)	24 (0.657)	6 (1.392)	17 (1.349)	19 (0.462)	7 (0.311)	4 (0.476)	1 (0.433)	54
Euphonia hirundinacea	56 (1.648)	47 (1.287)	0	135 (#####)	65 (1.580)	73 (3.243)	50 (5.956)	9 (3.893)	332
Cyanerpes cyanea	9 (0.265)	21 (0.575)	0	6 (0.476)		2 (0.089)	2 (0.238)	0	40
Chlorospingus ophthalmicus	2 (0.059)	2 (0.055)	1 (0.232)	80 (6.347)	4 (0.097)	5 (0.222)	2 (0.238)	0	96
Thraupis abbas	1 (0.029)	0	0	0	0	0	0	0	1
Thraupis episcopus	0	3 (0.082)	0	0	0	0	0	0	3
Eucometis penicillata	65 (1.913)	48 (1.315)	2 (0.464)	0	0	1 (0.044)	2 (0.238)	0	118

Species	(1)	(2)	(3)	(4)	(5)	(6)	(7)	(8)	Total
Lanio aurantius	15 (0.441)	14 (0.383)	1 (0.232)	4 (0.317)	2 (0.049)	6 (0.267)	0	1 (0.433)	14
Habia fuscicauda	204 (6.004)	90 (2.465)	28 (6.497)	26 (2.063)	12 (0.292)	17 (0.755)	36 (4.288)	11 (4.758)	424
Habia rubica	112 (3.296)	78 (2.136)	18 (4.176)	34 (2.697)	12 (0.292)	29 (1.288)	18 (2.144)	4 (1.730)	305
Saltator atriceps	1 (0.029)	1 (0.027)	0	0	0	0	0	0	2
Saltator maximus	0	7 (0.192)	2 (0.464)	2 (0.159)	3 (0.073)	0	4 (0.476)	0	6
Caryothraustes poliogaster	7 (0.206)	11 (0.301)	10 (2.320)	6 (0.476)	7 (0.170)	11 (0.489)	1 (0.119)	0	53
Cyanocompsa cyanoides	34 (1.001)	21 (0.575)	2 (0.464)	3 (0.238)	7 (0.170)	8 (0.355)	4 (0.476)	0	79
Cyanocompsa parellina	19 (0.559)	22 (0.603)	0	13 (1.031)	11 (0.267)	16 (0.711)	24 (2.859)	0	64
Arremonops rufivirgatus	1 (0.029)	5 (0.137)	0	5 (0.397)	5 (0.122)	3 (0.133)	0	2 (0.865)	16
Volatinia jacarina	2 (0.059)	131 (3.588)	0	13 (1.031)	0	16 (0.711)	0	0	162
Tiaris olivaceus	11 (0.324)	42 (1.150)	4 (0.928)	23 (1.825)	5 (0.122)	10 (0.444)	1 (0.119)	1 (0.433)	44
Sporophila torqueola	0	0	0	6 (0.476)	2 (0.049)	39 (1.733)	2 (0.238)	1 (0.433)	50
Aimophila rufescens	0	0	0	2 (0.159)	1 (0.024)	0	0	0	3
Molothrus aeneus	1 (0.029)	0	0	0	0	0	0	0	1
Dives dives	0	0	0	0	0	0	0	2 (0.865)	2

Species	(1)	(2)	(3)	(4)	(5)	(6)	(7)	(8)	Total
Amblycercus holosericeus	0	15 (0.411)	1 (0.232)	5 (0.397)	1 (0.024)	0	1 (0.119)	0	23
Icterus dominicensis	0	0	0	0	0	1 (0.044)	0	0	1
