# Supplementary figures and images for: Decadal changes and delayed avian species losses due to deforestation in the northern Neotropics
Source: PeerJ. 2013 Oct 8;1:e179. doi: 10.7717/peerj.179 (PMC3796372; doi:10.7717/peerj.179)

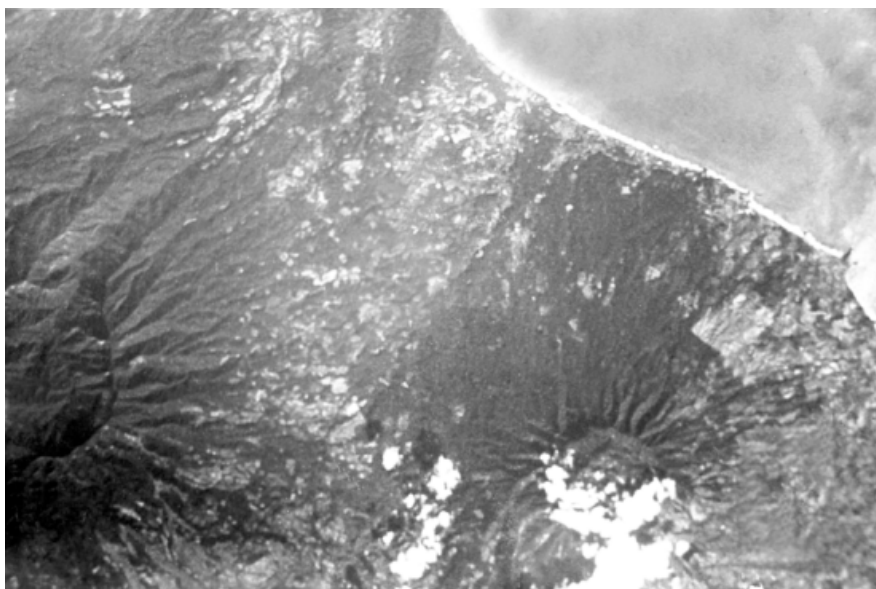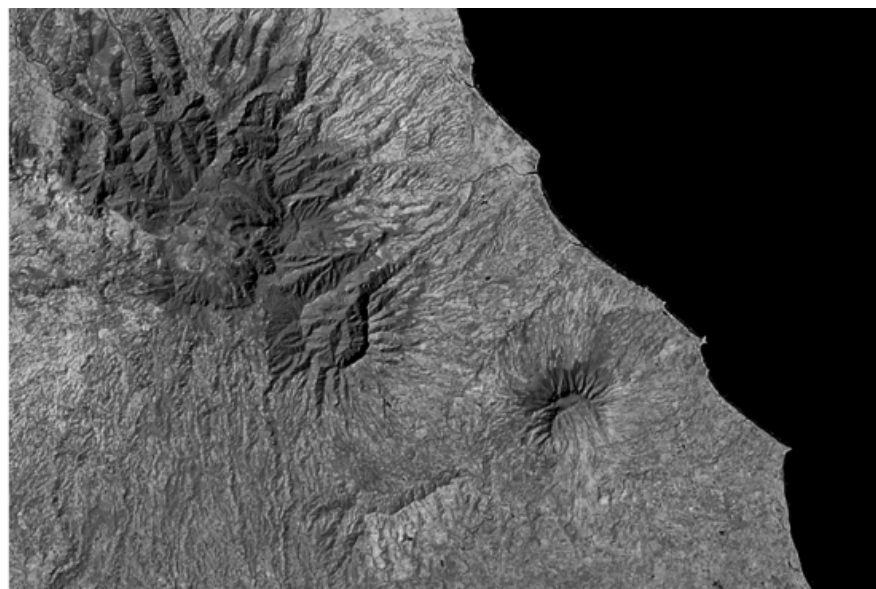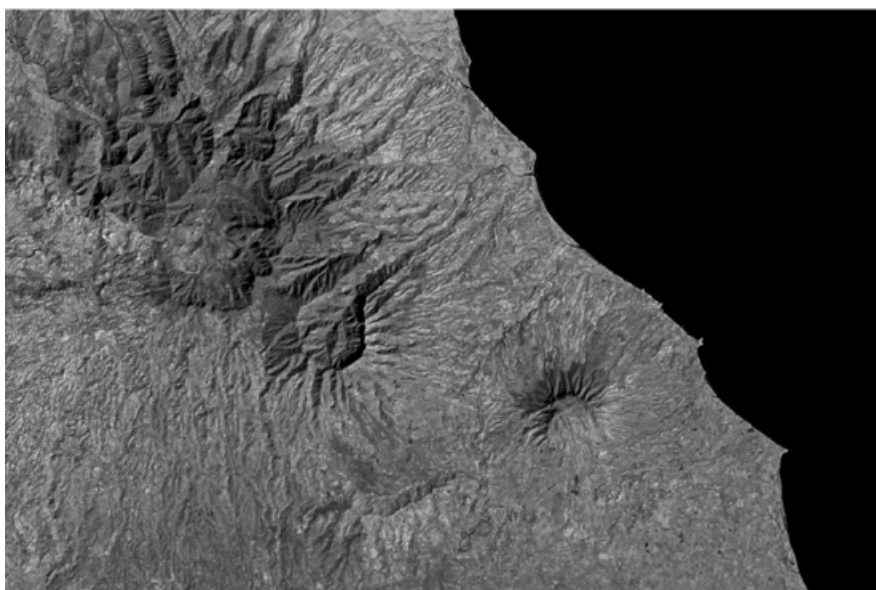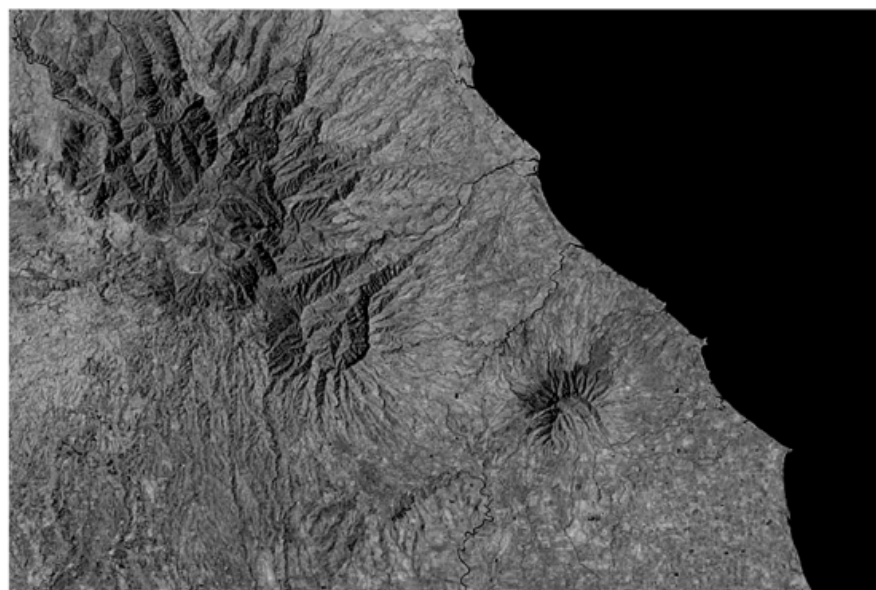

1  
2 Fig. S1

Supplement: Figure S1 — A series of satellite images depicting deforestation in Los Tuxtlas, focusing in on the volcanoes Santa Marta (left) and San Martín Pajápan (right), starting with a 1973/4 Skylab image (upper left) and progressing through a series of Landsat images, from 1999 (upper right), 2003 (lower left), and 2011 (lower right). [file peerj-01-179-s002.pdf]

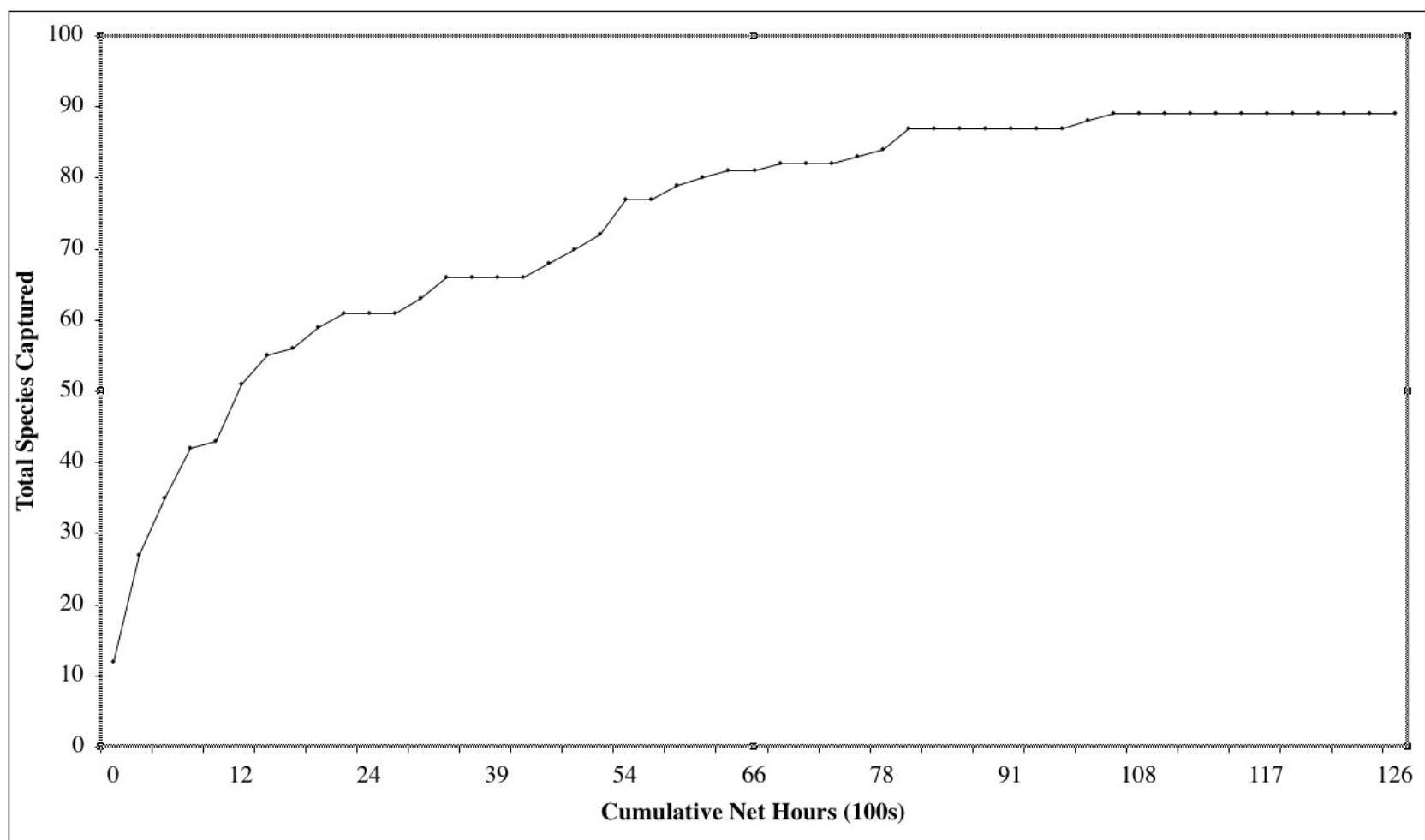

Fig. S2

Supplement: Figure S2 [file peerj-01-179-s003.pdf]
